# Editorial: Current advances in the metabolism of mycotoxins in plants

**DOI:** 10.3389/fpls.2023.1343855

**Published:** 2023-12-04

**Authors:** Giovanni Beccari, Francesco Tini, Hans J. L. Jørgensen

**Affiliations:** ^1^ Department of Agricultural, Food and Environmental Sciences, University of Perugia, Perugia, Italy; ^2^ Department of Plant and Environmental Sciences, Faculty of Science, University of Copenhagen, Frederiksberg, Denmark

**Keywords:** secondary metabolites, fungi, cereals, bean, *Fusarium*, *Aspergillus*, *Trichoderma*

Mycotoxins are toxic secondary metabolites (SM) produced by various genera of filamentous fungi. They can induce a wide variety of harmful effects in humans and animals. Often, mycotoxin accumulation begins in plants when they are attacked by mycotoxigenic fungi. However, plants have the ability to transform and detoxify phytotoxic chemical compounds such as mycotoxins (Berthiller et al., 2007; Berthiller et al., 2013). Thus, plants can transform mycotoxins, and in some cases, the resulting compounds may be harmless, while in others, they may be toxic, being considered as “masked mycotoxins” (Freire and Sant’Ana, 2018; Zhang et al., 2020). For some mycotoxins, the host’s ability to metabolize these compounds is well-known, while for others, limited data are available. Conversely, SM produced by non-pathogenic fungal microorganisms could have an antifungal role against pathogenic fungi (Rodrigo et al., 2022). SM can also interact with plant metabolism, enhancing the plant’s defence to counteract pathogens (Elhamouly et al., 2022).

For all these reasons, understanding the metabolism of mycotoxins in plants is a key aspect of counterbalancing their spread in food/feed products and adopting mitigation tactics to reduce human and animal health hazards. In this context, the objective of this Research Topic was to collect articles relative to the current advances in the metabolism of mycotoxins. This Research Topic contains five manuscripts that cover significant aspects of this field.

The paper written by Meng et al. highlights introductory elements showing the presence of mycotoxigenic fungi in cereal grain. They reported the presence of *Aspergillus*, *Fusarium* and *Alternaria*, as well as their abilities to produce mycotoxins, in wheat and paddy grains (fresh and stored samples) collected in 2021 from Shanghai (China). *Fusarium sambucinum* and species within *Aspergillus* section *flavi* were more frequently found in paddy grains, whereas *Alternaria* was the predominant genus in wheat. *Fusarium* spp. were the main species in fresh grains and *Aspergillus* was predominant in stored grains. *In vitro* assessments on potato dextrose agar demonstrated that *Fusarium* isolates were mainly capable of producing deoxynivalenol and zearalenone. Aflatoxins were exclusively produced by *Aspergillus* section *flavi*. *Alternaria* species predominantly synthesized alternariol, together with other mycotoxins like alternariol monomethyl ether, altenuene, tenuazonic acid, tentoxin, and altenusin. A combined analysis of the mycobiota and their mycotoxin-producing potential provides valuable insights into mycotoxin-production mechanisms, thereby facilitating the development of effective prevention and control strategies for wheat and paddy in a specific geographic region.

In addition to paddy and wheat grains, *Aspergillus* section *flavi* is also often associated with maize grains causing aflatoxins contaminations (Smith et al., 2019). In this context, the paper by Castano-Duque et al. provides interesting discoveries on the modulation by flavonoids of aflatoxins accumulation from *Aspergillus flavus* in maize kernels. To understand the molecular mechanisms of maize resistance to *A. flavus* colonization and aflatoxin accumulation better, they performed a transcriptomic study in maize kernels infected by the fungus. They found a significant association between flavonoid biosynthetic pathway genes and infection by *A. flavus*. This suggests that flavonoids can contribute to host resistance mechanisms against aflatoxin contamination through the modulation of toxin accumulation in maize kernels.

Maize grains can also be colonized by other mycotoxigenic species such as those belonging to the genus *Fusarium* (Bryła et al., 2022). In this context, Cao et al. investigated the resistance of maize kernels to Fusarium ear rot (FER), caused by *Fusarium verticillioides* as well as fumonisin accumulation. For this, quantitative trait loci (QTLs) and bulk-segregant RNA-seq techniques were utilized. Genetic regions within genetic bins 4.07-4.1, 6-6.01, 6.04-6.05, and 8.05-8.08 were linked to FER resistance and reduced fumonisin levels. Transcriptome comparisons between resistant and susceptible inbred bulks at 10 days post-inoculation with *F. verticillioides* identified 364 differentially expressed genes (DEGs). In resistant bulks, genes associated with fatty acid and starch biosynthesis, cell division and phytosulfokine signalling were downregulated. Conversely, genes tied to secondary metabolism, cell wall biosynthesis/rearrangement and flavonoid production were upregulated, reflecting a growth-defence trade-off. Several DEGs, located within QTL confidence intervals and showing significant expression differences between resistant and susceptible bulks, were identified as candidate genes for FER resistance and fumonisin reduction.

Studying the same host-pathogen interaction, Cao et al. performed a metabolome study in maize, comparing recombinant inbred lines with and without inoculation with *F. verticillioides.* The maize lines varied in resistance to the pathogen and the aim was to elucidate pathways involved in resistance and find markers for FER and fumonisin contamination in maize kernels. Data obtained 10 days after inoculation explained differences between resistance and susceptibility better than data from 3 days. It was found that differences in membrane lipid homeostasis, methionine metabolism and modulation of indolacetic acid conjugation appeared relevant when distinguishing between resistant and susceptible lines. Furthermore, specific metabolites, such as polyamine spermidine and the alkaloid isoquinoline appeared to be interesting when looking for improvement of resistance to FER and reduction of fumonisin levels.

SM produced by microorganisms may have an antifungal role against phytopathogenic fungi. Among different microorganisms, the species of the genus *Trichoderma* are the most potent biocontrol agents in use today, among others because they produce a diverse range of antimicrobial SM (Khan et al., 2020). Cardoza et al. studied the biocontrol fungus *Trichoderma arundinaceum*, which has the potential as a biological control agent, but also can produce trichothecene toxins. Three *Trichoderma arundinaceum* isolates from bean fields produced trichothecene harzianum A (HA) and trichodermol (an intermediate in the HA-biosynthesis). The three isolates showed antifungal activity against the bean pathogens *Rhizoctonia solani* and *Sclerotinia sclerotiorum in vitro* and this was ascribed to HA production. Furthermore, seed treatment with the *Trichoderma*-isolates gave faster germination and, in some cases, increased growth of aerial parts of bean plants. Transcriptomics of bean plants inoculated with the *T. arundinaceum* isolates indicated that HA production increased the expression of plant defence-related genes, especially chitinase-encoding genes. Collectively, the study indicated that *Trichoderma* species can induce resistance in plants without negative effects on plant growth.

The collection of articles on this Research Topic provides interesting information on the presence of mycotoxins in plants, the plant’s activity to reduce the accumulation of mycotoxins, as well as the interaction of some SM with the host, enhancing its ability to counteract pathogens.

## Author contributions

GB: Conceptualization, Writing – original draft, Writing – review & editing. FT: Conceptualization, Writing – original draft, Writing – review & editing. HJ: Conceptualization, Writing – original draft, Writing – review & editing.
